# Long-term effectiveness of banning highly hazardous pesticides on suicide mortality: a 12-year quasi-experimental study in South Korea

**DOI:** 10.1017/S2045796026100705

**Published:** 2026-05-28

**Authors:** Yangwoo Kim, Inah Kim, Jeehee Min, Soo-Jin Lee

**Affiliations:** 1 Department of Social and Preventive Medicine, Inha University College of Medicine, Incheon, Republic of Korea; 2 Department of Occupational and Environmental Medicine, Inha University Hospital, Incheon, Republic of Korea; 3 Department of Occupational and Environmental Medicine, Hanyang University College of Medicine, Seoul, Republic of Korea; 4 Graduate School of Public Health, Hanyang University, Seoul, Republic of Korea; 5 Department of Occupational and Environmental Medicine, Hanyang University Hospital, Seoul, Republic of Korea

**Keywords:** highly hazardous pesticides, interrupted time series analysis, paraquat, pesticides, Republic of Korea, suicide

## Abstract

**Aims:**

Pesticide self-poisoning accounts for 14–20% of global suicides, predominantly in agricultural regions where highly hazardous pesticides (HHPs) remain accessible. This poses a critical challenge in South Korea despite its advanced healthcare system. In response, South Korea implemented a phased ban on HHPs, including paraquat, beginning in November 2011. This study aimed to evaluate the long-term effect of this ban on pesticide suicide mortality.

**Methods:**

We conducted an interrupted time series study using national mortality data from 2004 to 2023, with autoregressive integrated moving average errors to account for autocorrelation. Sensitivity analyses included alternative intervention timings, period definitions and outcome measures. The primary outcome was the monthly count of pesticide-related suicides.

**Results:**

Among 268,869 suicide deaths recorded during the study period, 34,962 (13.0%) involved pesticide poisoning. The monthly pesticide suicide counts declined from a mean of 244 before the ban to 81 afterwards. The interrupted time series analysis revealed no immediate level change following the ban (−0.204; 95% confidence interval [CI]: −0.989 to 0.581; *P* = 0.61) but showed a significant acceleration in the decline during the first 36 months (initial slope change: −0.122; 95% CI: −0.163 to −0.082; *P* < 0.001). Counterfactual projections estimated 10,846 deaths averted (95% CI: −567 to 29,715), representing a 48% model-based reduction. These effects were robust across all sensitivity analyses. Structural breakpoint analysis identified an increase in gas poisoning suicides in August 2008, more than three years before the ban, indicating no evidence of method substitution. The demographic profiles of pesticide and gas poisoning suicides differed substantially.

**Conclusions:**

The ban on HHPs in South Korea was associated with a sustained decline in pesticide suicide mortality over 12 years, with no evidence of method substitution. These findings support the means restriction through pesticide regulation as an effective suicide prevention strategy.

## Background

Suicide remains a major global public health challenge, accounting for over 700,000 deaths annually and ranking as the fourth leading cause of death among those aged 15–29 worldwide (Pirkis *et al.*, 2024; GBD 2021 Suicide Collaborators, 2025). Among suicide methods, pesticide self-poisoning predominates in low- and middle-income countries, where it accounts for an estimated 14–20% of all suicide deaths globally (Gunnell *et al.*, 2017; Mew *et al.*, 2017). The high case-fatality rate associated with highly hazardous pesticides (HHPs) makes this method especially lethal, given pre-hospital mortality (Konradsen *et al.*, 2007).

Means restriction – limiting access to lethal suicide methods – is an evidence-based strategy for suicide prevention (Arensman *et al.*, 2020; Pirkis *et al.*, 2024). Suicidal crises are often transient; therefore, reducing access to highly lethal means can prevent deaths (Pirkis *et al.*, 2024). International evidence indicates that national HHP bans are associated with substantial reductions in pesticide-related suicides without complete substitution (Gunnell *et al.*, 2017; Rubbo *et al.*, 2025). Studies from Sri Lanka and China reported notable declines following pesticide regulations (Knipe *et al.*, 2014; Yan *et al.*, 2023; Noghrehchi *et al.*, 2024).

While pesticide-related suicides are most common in low- and middle-income countries, South Korea – with its extensive agricultural sector and easy access to pesticides – has experienced substantial pesticide-related mortality. The country ranks among the Organisation for Economic Co-operation and Development nations with the highest suicide rates, with age-standardized figures exceeding 20 per 100,000 people in recent years (Organisation for Economic Co-operation and Development, 2025). Historically, pesticide ingestion was a prevalent suicide method, particularly in rural regions (Ko *et al.*, 2018; Cha *et al.*, 2019). In 2011, South Korea implemented a phased paraquat ban, following European Union regulatory decisions and concerns about neurotoxicity and Parkinson’s disease (Vaccari *et al.*, 2019). Although suicide prevention was not the primary objective, restricting this highly lethal, antidote-lacking pesticide could reduce suicide mortality. Sales restrictions began in November 2011, followed by a complete ban in October 2012 (Cha *et al.*, 2016a). Several other highly toxic insecticides – including organophosphates and endosulfan, classified as WHO Class I–II hazards – were concurrently banned in December 2011 (Lin *et al.*, 2026). Although category-specific mortality data were unavailable, prior research indicated paraquat accounted for the majority of pesticide suicide deaths (Cha *et al.*, 2016a). An initial assessment reported a 37% decrease in pesticide suicides in 2013 compared with rates expected from pre-ban trends (Cha *et al.*, 2016a).

However, existing studies evaluating the Korean paraquat ban have notable limitations. The initial analysis by Cha *et al.* (2016a) extended only two years after the ban (up to 2013), limiting assessment of long-term effects and potential method substitution. More recent analyses extended observations to 2022 but primarily employed joinpoint regression (Choi *et al.*, 2026). While joinpoint regression effectively identifies changes in trends, it cannot distinguish policy effects from concurrent secular influences. This limitation is salient as the paraquat ban coincided with confounding factors – the 2008 global financial crisis aftermath and high-profile celebrity suicides – that elevated suicide rates during 2009–2011 (Chan *et al.*, 2014; Chen *et al.*, 2014). Quasi-experimental approaches incorporating comparison groups can better isolate policy effects from such concurrent changes. Although previous studies observed no overall increase in non-pesticide suicides, the temporal relationship between the decline in pesticide suicides and shifts in alternative methods remains insufficiently characterized.

This study aimed to evaluate the effect of the paraquat ban on pesticide suicide mortality in South Korea using national mortality data spanning 2004–2023, providing over a decade of post-ban follow-up. We applied interrupted time series (ITS) analysis with autoregressive integrated moving average (ARIMA) errors as the primary approach to characterize changes in pesticide suicide trends. As an exploratory analysis, we also conducted difference-in-differences (DiD) comparisons using non-pesticide suicide methods to provide supplementary evidence regarding the policy effect. We hypothesized that the ban would accelerate pesticide suicide decline, manifesting as a steeper downward slope rather than an abrupt level change, reflecting gradual stock depletion after sales restrictions. We also examined potential method substitution through breakpoint analysis to determine whether changes in alternative suicide methods temporally coincided with the pesticide ban.

## Methods

### Study design and setting

This ITS study with a DiD extension assessed the impact of South Korea’s paraquat ban on suicide mortality (Bernal *et al.*, 2017; Lopez Bernal *et al.*, 2018). The study period spanned January 2004 to December 2023, encompassing 95 pre-intervention and 145 post-intervention months. It began in January 2004, after pesticide suicide rates peaked and trends shifted from rapid increase to gradual decline, to ensure a stable baseline (Cha *et al.*, 2019). Mortality data were extracted in July 2025 following approval from the institutional review board.

### Data sources

Mortality data were obtained from the Microdata Integrated Service of Statistics Korea (https://mdis.kostat.go.kr), a national vital registration system with >99% completeness (Cha *et al.*, 2016b). Causes of death were coded using the Korean Standard Classification of Diseases, based on the International Classification of Diseases, 10th Revision (ICD-10). Individual records included the date of death, underlying cause of death, sex, age, area of residence and occupation. Occupation was categorized as recorded, with missing or unspecified cases classified as ‘economically inactive or unspecified’. Mid-year population estimates by sex and five-year age groups were obtained from Statistics Korea; for monthly rate calculations, population denominators were obtained by linearly interpolating between adjacent years’ mid-year estimates to avoid artificial discontinuities at year boundaries.

### Study population

All residents of South Korea aged ≥15 years who died by suicide during the study period were included. Deaths among those under 15 were excluded due to low counts and distinct etiological patterns.

### Outcome definitions

Suicide deaths were identified using ICD-10 codes for intentional self-harm (X60–X84), with three primary outcome categories: (1) pesticide suicide (intentional self-poisoning by pesticides, X68); (2) hanging suicide (hanging, strangulation, or suffocation, X70); and (3) total suicide (all methods, X60–X84). For the DiD analyses, non-pesticide suicides were calculated by subtracting pesticide suicides from total suicides.

### Intervention

The Korean government implemented a phased ban on paraquat – the most common pesticide for self-poisoning (case-fatality rate >70%) (Cha *et al.*, 2016a). The Rural Development Administration cancelled paraquat re-registration on 23 November 2011, restricting new sales and a complete ban took effect on 31 October 2012. The intervention point was defined as December 2011, the first full month following the policy announcement (23 November 2011), to capture behavioural responses from the earliest stage of public awareness.

### Statistical analysis

#### Primary analysis: ITS

We modelled monthly pesticide suicide counts using an ITS regression with ARIMA errors (Hyndman and Khandakar, 2008):

$$Y_{t}=\beta_{0}+\beta_{1}\cdot time_{t}+\beta_{2}\cdot level_{t}+\beta_{3}\cdot slope_{initial_{t}}+\beta_{4}\cdot slope_{later_{t}}+N_{t}$$

where *Y_t_* represents the transformed suicide count at month *t*; time*_t_* denotes months since the study start; level*_t_* is an indicator for the post-intervention period; slope_initial*t*_ captures the trend change during the initial post-ban period (months 1–36); slope_later*t*_ represents the trend change during the later post-ban period (month 37 onward); and *N_t_* follows an ARIMA process. The 36-month initial period was chosen to approximate the combined duration of regulatory phase-in and post-ban stock exhaustion. Given the phased implementation – sales restriction from November 2011 followed by a complete ban from October 2012 – the 36-month window encompasses an approximately 12-month regulatory transition plus an additional period for depletion of existing stocks (Gunnell *et al.*, 2017). Alternative durations (6, 12, 24, 48 and 60 months) were examined in sensitivity analyses (eTable 6). Both level change and slope changes were modelled; slope changes were expected to predominate given gradual stock depletion following the sales restriction.

For pesticide suicide counts, we applied a square root transformation based on Box–Cox optimization (*λ* = 0.358), which approximated the square root (*λ* = 0.5) and effectively stabilized the variance (eTable 1) (Box and Cox, 1964). Log transformation was also tested, but did not satisfy normality (Shapiro–Wilk *P* < 0.001 vs. *P* = 0.14 for square root). Sensitivity analysis confirmed consistent conclusions (eTable 7). For the DiD analyses, we used a logarithmic transformation to facilitate interpretation of interaction terms as relative changes, consistent with standard analytical practice (Angrist and Pischke, 2009). ARIMA order was selected via AICc with seasonal components (period = 12) (eTable 2; eFig. 1; eFig. 2) (Yang *et al.*, 2019). Model adequacy was evaluated using the Ljung–Box and Shapiro–Wilk tests (eFig. 3).

We reported results for both count-based and rate-based (age–sex-standardized mortality rates per 100,000) analyses, with Wald-based 95% confidence intervals (CIs) supported by residual diagnostics (Table 1). Block bootstrap resampling (1,000 replications; block length = 15 months, exceeding the seasonal period to preserve autocorrelation) provided a robustness check (Davison and Hinkley, 1997; Politis and White, 2004).

**Table 1.** Interrupted time series analysis of pesticide suicide following the paraquat ban in South Korea, 2004–2023 Table 1 long description.

Parameter	Wald estimate (95% CI)	*P*-value	Bootstrap 95% CI
Panel A: Count-based analysis (square-root scale)			
Level change	−0.204 (−0.989 to 0.581)	0.610	−0.645 (−4.125 to 2.317)
Initial slope change (months 1–36)	−0.122 (−0.163 to −0.082)	<0.001	−0.115 (−0.214 to 0.006)
Later slope change (months 37+)	0.012 (−0.012 to 0.036)	0.332	0.004 (−0.038 to 0.046)
Panel B: Rate-based analysis (ASR per 100,000)			
Level change	−0.012 (−0.047 to 0.023)	0.518	−0.030 (−0.213 to 0.102)
Initial slope change (months 1–36)	−0.005 (−0.007 to −0.003)	<0.001	−0.005 (−0.009 to 0.000)
Later slope change (months 37+)	0.001 (0.000–0.002)	0.034	0.001 (−0.001 to 0.003)

ASR, age–sex-standardized rate; CI, confidence interval. Model: ARIMA(1,0,2)(2,0,0)[12]. Level change: immediate shift at intervention (December 2011). Slope changes: trend change during initial (months 1–36) and later (months 37+) periods. Standard population: 2010 Korean census. See Figure 3 for visualization of rate-based results.

#### Subgroup analyses

Subgroup analyses were stratified by sex (male, female), age group (15–29, 30–49, 50–64 and ≥65 years), region (capital region [Seoul, Incheon, Gyeonggi Province] vs. non-capital region) and occupation (agriculture, forestry and fishery [AFF]; other occupations; economically inactive or unspecified). These stratifications reflect prior evidence of higher pesticide suicide prevalence among older adults, rural residents and agricultural workers (Cha *et al.*, 2019). Given multiple comparisons, results should be interpreted with caution.

#### Sensitivity analyses

Sensitivity analyses evaluated robustness: (1) testing alternative intervention points (May 2012 and December 2012; eTable 5); (2) varying the definition of the initial post-intervention period (6, 12, 24, 48 and 60 months; eTable 6); (3) specifying an a priori two-stage intervention model based on the known policy timeline – with December 2011 (onset of the sales restriction) as the first intervention point and November 2012 (onset of the complete ban) as the second – to test whether effects differed between stages (eTable 11; eFigure 7); and (4) fitting the ARIMA model using only pre-intervention data (January 2004–November 2011) to project the counterfactual forward (eTable 10; eFigure 5).

#### Deaths averted estimation

Deaths averted were estimated by comparing observed counts with counterfactual projections, constructed by setting intervention-related coefficients to zero while retaining the pre-intervention trend. Percentage reduction was calculated as (deaths averted/counterfactual total) × 100. CIs were derived from block bootstrap resampling (1,000 replications), with the full modelling procedure repeated for each replicate.

#### Supplementary analyses

To explore potential method substitution, we performed Bai–Perron structural breakpoint analysis (Bai and Perron, 2003) on gas poisoning suicides (X67), an accessible and highly lethal method that increased following a high-profile celebrity suicide in 2008 (Chen *et al.*, 2014). A breakpoint preceding the paraquat ban would suggest that changes in gas poisoning were unrelated to pesticide regulation (eTable 9; eFigure 10). We also compared the demographic characteristics (sex and age distribution) of pesticide and gas suicide decedents using chi-square tests to assess whether these methods attracted distinct populations (Table 2; eFigure 9). Additionally, we performed ITS analysis on total suicide mortality to examine whether the pesticide ban reduced overall suicide deaths beyond method-specific effects (eTable 12; eFigure 8). As ARIMA cannot control for concurrent factors affecting all methods, we conducted DiD analyses comparing pesticide suicides with hanging and non-pesticide suicides for robustness (eTable 13) (Wing *et al.*, 2018).

**Table 2.** Demographic characteristics of suicide deaths by pesticide poisoning and gas poisoning in South Korea, 2004–2023 Table 2 long description.

Category	Pesticide *n* (%)	Gas *n* (%)	*P*-value	Cramer’s *V*
Sex			<0.001	0.149
Male	23,839 (68.2)	21,842 (81.4)		
Female	11,114 (31.8)	4,999 (18.6)		
Age group			<0.001	0.548
15–39	2,702 (7.7)	10,927 (40.7)		
40–64	13,107 (37.5)	14,090 (52.5)		
≥65	19,144 (54.8)	1,824 (6.8)		
Region			<0.001	0.029
Capital	15,466 (44.2)	12,659 (47.2)		
Non-capital	19,487 (55.8)	14,182 (52.8)		

Nine pesticide and 21 gas poisoning deaths with missing demographic data were excluded. Cramer’s *V*: <0.1 negligible, 0.1–0.3 small, 0.3–0.5 medium and >0.5 large effect size.

#### Software

All analyses were conducted using R version 4.4.2 (R Core Team, 2025), employing the forecast package for time-series modelling. Statistical significance was set at two-sided *P* < 0.05.

## Results

### Descriptive statistics

During the study period (January 2004 to December 2023), 268,869 suicide deaths were recorded in South Korea over 240 months. The pre-intervention period (January 2004–November 2011; 95 months) included 106,386 deaths (mean: 1,120/month), and the post-intervention period (December 2011–December 2023; 145 months) included 162,483 deaths (mean: 1,121/month).

**Figure 1.**
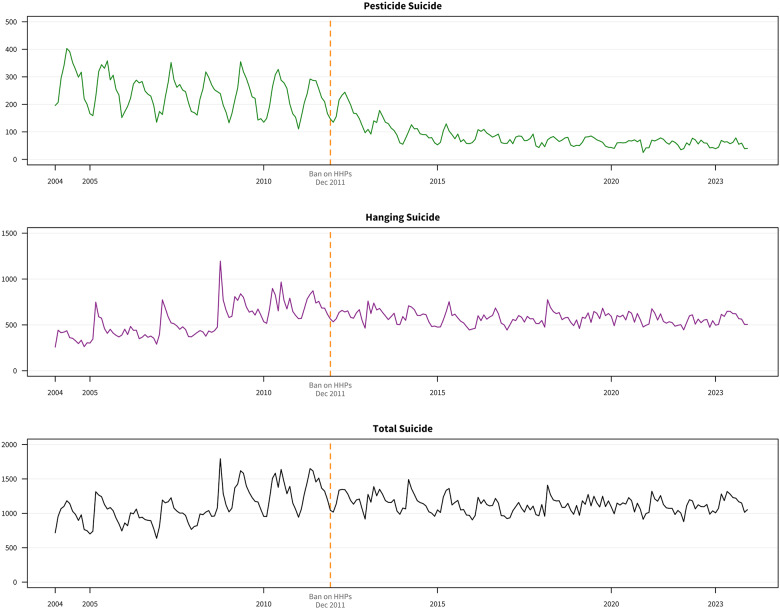
Monthly suicide counts by method in South Korea, 2004–2023. Figure 1 long description.

Figure 1 presents the monthly trends in suicide counts by method. Pesticide self-poisoning accounted for 34,962 deaths overall: 23,183 pre-intervention (mean: 244/month; 21.8% of suicides) and 11,779 post-intervention (mean: 81/month; 7.2%), a 67% reduction.

Monthly suicide counts for hanging (blue), pesticide poisoning (green), jumping (orange) and gas poisoning (red). Vertical dashed line: paraquat ban announcement (December 2011).

Before the intervention, the three most common suicide methods were hanging (48.9%), pesticide poisoning (21.8%) and jumping from height (14.1%). After the intervention, the ranking shifted to hanging (50.9%), jumping from height (16.4%) and gas poisoning (14.5%), with pesticide poisoning declining to fourth position (7.2%).

### Primary analysis: ITS

Table 1 summarizes the ITS analysis results for pesticide suicides, presenting both count-based (Panel A) and rate-based (Panel B; age–sex-standardized mortality rates per 100,000) analyses as primary estimates. All estimates are presented on the square-root scale. No significant immediate level change was detected following the intervention (−0.204; 95% CI: −0.989 to 0.581; *P* = 0.61). However, a significant initial slope change was observed during the first 36 months after the intervention (−0.122; 95% CI: −0.163 to −0.082; *P* < 0.001). No significant later slope change was detected from month 37 onward (0.012; 95% CI: −0.012 to 0.036; *P* = 0.33).

**Figure 2.**
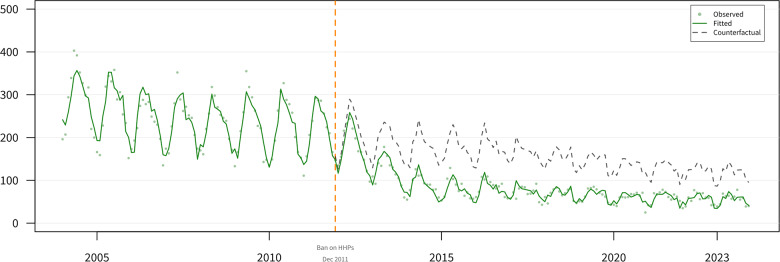
Interrupted time series analysis of pesticide-related suicides with counterfactual projection. Figure 2 long description.

**Figure 3.**
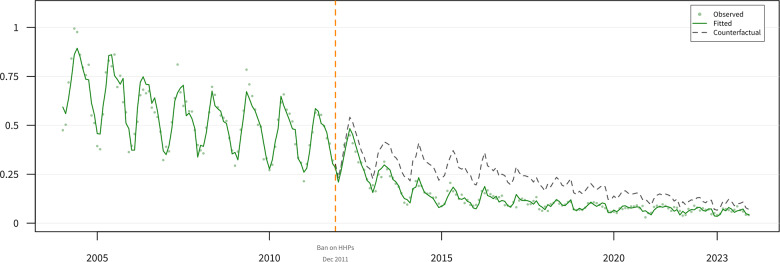
Age–sex-standardized pesticide suicide rates with counterfactual projection. Figure 3 long description.

The bootstrap CI for the initial slope change was wider (−0.115; 95% CI: −0.214 to 0.006). Figure 2 presents count-based results, and Figure 3 presents rate-based results.

Monthly pesticide suicide counts (green points) with fitted ARIMA model (green line) and counterfactual projection (grey dashed line). Vertical dashed orange line: paraquat ban (December 2011).

Age–sex-standardized pesticide suicide rates per 100,000 population. Green points: observed rates; green line: fitted values; grey dashed line: counterfactual projection. Standard population: 2010 Korean census. See eTable 4 for details.

To provide an interpretable effect size, we compared observed counts with counterfactual projections assuming no intervention. Over 144 post-intervention months, an estimated 10,846 pesticide suicide deaths were averted (95% bootstrap CI: −567 to 29,715), a 47.9% model-based reduction (95% CI: −5.1 to 71.6%) relative to the counterfactual. This estimate is more conservative than the descriptive 67% reduction because it accounts for pre-existing trends. The wide bootstrap CIs crossed zero, reflecting uncertainty in the effect magnitude.

### Subgroup analyses

eTable 3 and eFigure 4 present subgroup-specific results. All estimates correspond to initial period slope changes on the square-root scale; formal interaction tests were not performed, so subgroup differences are exploratory.

By sex, both males (−0.100; 95% CI: −0.137 to −0.064; *P* < 0.001) and females (−0.071; 95% CI: −0.095 to −0.047; *P* < 0.001) exhibited significant declines. By age, older adults aged 65 years and above showed the largest decline (−0.107; 95% CI: −0.136 to −0.078; *P* < 0.001), followed by adults aged 50–64 years (−0.060; 95% CI: −0.081 to −0.038; *P* < 0.001) and 30–49 years (−0.046; 95% CI: −0.070 to −0.021; *P* < 0.001). For adults aged 15–29 years, no significant decline was detected (0.006; 95% CI: −0.013 to 0.024; *P* = 0.55).

By region, non-capital regions experienced a numerically larger decline (−0.114; 95% CI: −0.151 to −0.076; *P* < 0.001) than the capital region (−0.048; 95% CI: −0.074 to −0.021; *P* < 0.001). By occupation, AFF workers (−0.065; 95% CI: −0.093 to −0.038; *P* < 0.001), those in other occupations (−0.044; 95% CI: −0.075 to −0.013; *P* = 0.005) and individuals with unspecified occupations (−0.093; 95% CI: −0.124 to −0.063; *P* < 0.001) all demonstrated significant declines.

Bootstrap CIs were wider for several subgroups, with partial discordance between Wald-based and bootstrap-based inferences in male, working-age and regional subgroups (eTable 3).

### Sensitivity analyses

eTables 4–7 present sensitivity analysis results. When age–sex-standardized mortality rates replaced counts (eTable 4), the initial slope change remained significant (−0.005; 95% CI: −0.007 to −0.003). With alternative intervention timing definitions (eTable 5), effect size decreased with later assumed intervention points: December 2011 (primary: −0.122; 95% CI: −0.163 to −0.082), May 2012 (+6 months: −0.085; 95% CI: −0.125 to −0.045) and December 2012 (+12 months: −0.043; 95% CI: −0.090 to 0.003), with the effect no longer significant at +12 months.

With alternative initial period lengths (eTable 6), significant declines were observed across all specifications (6, 12, 24, 48 and 60 months), with effect magnitude decreasing progressively with longer period definitions.

With log transformation (eTable 7), the initial slope change was −0.023 (95% CI: −0.029 to −0.018; *P* < 0.001), corresponding to a 2.3% monthly reduction (57% cumulative over 36 months). The direction and statistical significance of this result were consistent with the primary analysis. When the ARIMA model was fit using only pre-intervention data and projected forward, the estimated deaths averted were 11,473 (95% CI: 793–26,700), consistent with the primary analysis (eTable 10; eFigure 5). In the a priori two-stage intervention model, the first stage (December 2011 sales restriction) showed a significant slope change (−0.223; 95% CI: −0.395 to −0.051; *P* = 0.011), while the second stage (November 2012 complete ban) did not demonstrate an additional significant level change (−0.953; 95% CI: −2.08 to 0.176; *P* = 0.098) (eTable 11; eFigure 7). The absence of a significant additional effect in the second stage suggests that the primary impact on pesticide suicide mortality occurred during the first phase of pesticide restriction, when paraquat and several organophosphate insecticides were concurrently banned.

### Supplementary analyses

eTable 8 presents suicide method frequencies before and after the intervention. Pesticide poisoning deaths decreased from 23,183 (21.8%) to 11,779 (7.2%). Gas poisoning deaths increased from 3,300 (3.1%) to 23,562 (14.5%). Hanging deaths rose from 52,038 (48.9%) to 82,723 (50.9%), and jumping from height from 15,001 (14.1%) to 26,716 (16.4%).

eTable 9 and eFigure 10 summarize the structural breakpoint analysis for gas poisoning suicides. The first breakpoint was detected in August 2008, when the monthly mean count rose from 33 to 141.

ITS analysis of overall suicide counts (eTable 12; eFigure 8) showed significant initial (−0.007; 95% CI: −0.013 to −0.001; *P* = 0.017) and later slope changes (−0.004; 95% CI: −0.007 to −0.001; *P* = 0.006), with no immediate level change.

To examine the plausibility of method substitution, we compared demographic profiles of pesticide and gas poisoning suicides (Table 2; eFigure 9). Nine pesticide and 21 gas poisoning deaths with missing demographic data were excluded from this comparison. The two methods showed distinct age distributions: 54.8% of pesticide suicides occurred among adults aged 65 years and above (19,144/34,953) compared with 6.8% (1,824/26,841) for gas poisoning (Cramer’s *V* = 0.55). Sex distributions also differed, with males comprising 68.2% (23,839/34,953) of pesticide suicides versus 81.4% (21,842/26,841) of gas poisoning deaths (Cramer’s *V* = 0.15), whereas regional distributions were similar (Cramer’s *V* = 0.03).

As an exploratory analysis, we conducted DiD comparing pesticide suicides with hanging and non-pesticide suicides (eTable 13; eFigure 6). Although the initial slope change was significant in both comparisons (vs. hanging: −0.026; 95% CI: −0.040 to −0.012; vs. non-pesticide: −0.028; 95% CI: −0.041 to −0.015), the parallel trend assumption was violated. These results should be interpreted with caution as supportive evidence.

## Discussion

In this ITS study using national mortality data from South Korea, we found that the paraquat ban was associated with a significant decline in pesticide suicide mortality during the first three years following its implementation. This effect manifested as a steeper monthly decline rather than an immediate level change, consistent with gradual stock depletion following the sales ban. The pattern was robust across sensitivity analyses with alternative intervention timings, period definitions and outcome measures. We found no evidence of method substitution: gas poisoning increases preceded the ban by over three years, and demographic profiles of pesticide and gas suicide decedents differed substantially.

Our study extends prior research on Korea’s paraquat ban. Cha et al. reported a 37% decline two years post-ban (Cha *et al.*, 2016a); our decade-long follow-up revealed even greater reductions, suggesting sustained effects beyond initial evaluation. A recent segmented regression analysis extending to 2019 reported 15% and 36% step reductions in pesticide suicide rates in 2012 and 2013, respectively, with an estimated 8,353 suicides averted (Lin *et al.*, 2026). This is consistent with global evidence: Sri Lanka’s phased pesticide bans resulted in approximately a 50% reduction in suicide mortality (Knipe *et al.*, 2014; Bandara *et al.*, 2024; Noghrehchi *et al.*, 2024), and China experienced similar declines following HHP regulations (Yan *et al.*, 2023). In high-income Asian countries, Japan’s paraquat restrictions were associated with a 92% reduction in pesticide deaths over 33 years (Eddleston *et al.*, 2022), and Taiwan’s 2018 paraquat ban was followed by a 37% reduction in pesticide suicides (Chang *et al.*, 2022). Systematic reviews confirm that national HHP bans reduce pesticide suicides without full method substitution (Gunnell *et al.*, 2017; Rubbo *et al.*, 2025), particularly benefiting young individuals and females in LMICs (Schölin *et al.*, 2023).

The observed pattern of effects aligns with the mechanism of means restriction. Paraquat’s exceptional lethality (case-fatality >70%, no antidote) (Cha *et al.*, 2016a) means restricting access prevents substitution-resistant deaths. The absence of an immediate level change, followed by a steeper decline over the subsequent three years, reflects the gradual depletion of existing paraquat stocks after the sales ban. The phased regulatory timeline – sales restriction in November 2011, followed by a complete ban in October 2012 – implies approximately 12 months of continued limited availability, suggesting stock exhaustion within 2–3 years, making the 36-month initial period a reasonable specification confirmed by consistent effects across alternative definitions ranging from 6 to 60 months (eTable 6). Subgroup findings reinforce this mechanism, as larger effects were observed among older adults and rural residents – groups with historically higher accessibility to pesticides (Cha *et al.*, 2019). The consistent direction of effects across all subgroups provides the primary evidence for effect heterogeneity. Effect magnitude diminished with later intervention points, becoming non-significant at +12 months (December 2012) – consistent with policy impact depending on stock exhaustion timing. The a priori two-stage intervention model, specified according to the known policy timeline, revealed a significant slope change following the first-stage restriction (December 2011) but no additional significant effect following the second-stage complete ban (November 2012; eTable 11), suggesting that the initial restriction drove the primary reduction while the subsequent complete ban sustained rather than amplified the effect. The concurrent removal of several organophosphate insecticides during the first phase may have amplified this effect, although paraquat likely accounted for most of the reduction given its disproportionate case-fatality rate (Cha *et al.*, 2016a).

Our analysis found no evidence supporting method substitution. The breakpoint analysis identified August 2008 – approximately 40 months before the paraquat ban – as the structural change point for gas poisoning suicides, preceding a widely publicized celebrity suicide by charcoal burning in October 2008 by about two months (Chen *et al.*, 2014). This discrepancy may reflect algorithm sensitivity or gradual diffusion before the event. Regardless, the structural increase in gas poisoning clearly preceded the paraquat ban by more than three years, indicating that pesticide suicide declines and gas poisoning increases were independent phenomena. Lin *et al.* (2026) independently confirmed that gas poisoning increases commenced in 2008 and did not intensify following the pesticide bans. This temporal dissociation aligns with evidence that complete method substitution following means restriction is uncommon (Gunnell *et al.*, 2017). The demographic profiles of pesticide and gas poisoning suicides differed substantially (Table 2): pesticide suicides were concentrated among older adults, whereas gas poisoning was more prevalent among younger age groups, suggesting largely non-overlapping populations at risk and making direct substitution unlikely. Overall suicide mortality showed significant declines in both initial and later periods (eTable 12), suggesting pesticide suicide reductions contributed to overall mortality decline and substitution was incomplete.

As an exploratory analysis, we conducted DiD comparisons with hanging and non-pesticide suicides. Although parallel trends were violated, significant DiD estimates provide supplementary evidence that pesticide-specific declines exceeded secular trends, albeit requiring cautious interpretation.

This study has several strengths. First, the 12-year post-ban follow-up – the longest Korean paraquat ban evaluation – enables assessment of both initial acceleration and long-term sustainability. Second, combining ITS with DiD analysis provides stronger causal inference than conventional trend analyses (Bernal *et al.*, 2017; Wing *et al.*, 2018). Third, extensive sensitivity analyses – varying intervention timing, period definitions, outcome measures, transformation methods and counterfactual projections – consistently supported the primary findings.

The bootstrap CIs were wider than Wald-based intervals and included zero for the primary effect estimate, reflecting conservatism when autocorrelation is already modelled by ARIMA. Given adequate model specification (Ljung–Box *P* = 0.43), we report Wald-based inference as primary. The point estimate of approximately 10,800 deaths averted should be considered alongside the substantial uncertainty reflected in these wider bootstrap intervals.

Several limitations should be acknowledged. First, as an ecological study relying on aggregate data, we cannot draw individual-level causal inferences. Second, the parallel trends assumption underlying the DiD analysis was violated: pesticide suicides declined significantly faster than comparison methods prior to the intervention (slope difference ≈ −0.01 per month; *P* < 0.001). Methodological literature emphasizes that transparent reporting of pre-trend violation direction and magnitude – rather than discarding the DiD approach – allows valid contextual interpretation (Roth, 2022; Rambachan and Roth, 2023). Pesticide suicides were already declining more rapidly than comparison groups before the ban, likely reflecting rapid urbanization and the decline of the agricultural workforce, reducing the at-risk population. Because these shifts exerted continuous downward pressure independent of policy, the significant DiD decline suggests the ban achieved additional effects beyond the pre-existing trajectory. Thus, violation of the parallel trends assumption implies that our findings likely represent a conservative estimate of the true policy impact. Third, concurrent initiatives – such as the March 2011 Suicide Prevention Act establishing national coordination, hotlines and media guidelines – may have contributed to overall mortality decline. However, because this legislation did not include pesticide-specific measures, and the DiD framework adjusts for factors affecting all suicide methods, it is unlikely to account for the pesticide-specific effects observed in this study.

The sustained decline underscores means restriction as an effective public health strategy, reinforcing WHO/FAO recommendations to regulate HHPs (World Health Organization and Food and Agriculture Organization of the United Nations, 2023; Pirkis *et al.*, 2024). Economic modelling confirms HHP bans are cost-effective (Lee *et al.*, 2021). Given that pesticide self-poisoning contributes 14–20% of global suicides (Mew *et al.*, 2017), our findings may inform policy where HHPs remain available. Future studies should examine non-fatal poisoning and regulations targeting other hazardous compounds.

The ban on HHPs in South Korea was associated with a sustained decline in pesticide-related suicide mortality over a 12-year period, with no evidence of method substitution. These findings reinforce the means restriction through pesticide regulation as an effective and evidence-based component of comprehensive national suicide prevention strategies.

## Supporting information

10.1017/S2045796026100705.sm001 Kim et al. supplementary material Kim et al. supplementary material

## Data Availability

The data used in this study were obtained from the Microdata Integrated Service (MDIS) of Statistics Korea (https://mdis.kostat.go.kr). Access to the data is available upon request through the MDIS portal, subject to approval by Statistics Korea.

## Supplementary material

The supplementary material for this article can be found at https://doi.org/10.1017/S2045796026100705.

## Table 1 Long description

The table analyzes the impact of the paraquat ban on pesticide suicide rates in South Korea from 2004 to 2023, using count-based and rate-based analyses. In the count-based analysis, there was a non-significant immediate level change, but a significant reduction in the initial slope over the first 36 months. The later slope change was not significant. In the rate-based analysis, the immediate level change was also non-significant, but the initial slope showed a significant decrease, indicating a reduction in suicide rates per 100,000 people. The later slope change was small but statistically significant. These findings suggest that the ban had a more pronounced effect in the initial years, with diminishing changes over time.

Navigate back to Table 1.

## Table 2 Long description

The table compares demographic characteristics of suicide deaths by pesticide and gas poisoning in South Korea from 2004 to 2023. Males account for a higher percentage of deaths in both categories, with gas poisoning showing a larger male predominance. Age distribution reveals that younger individuals are more affected by gas poisoning, whereas older individuals are more likely to die from pesticide poisoning. Regional differences are negligible, with similar proportions of deaths occurring in capital and non-capital areas for both poisoning types. The effect size for age group differences is large, indicating a significant disparity in age-related trends between the two poisoning methods.

Navigate back to Table 2.

## Figure 1 Long description

The image contains three line graphs. The first graph shows 'Pesticide Suicide' trends from 2004 to 2023, with a noticeable decline after 2011. The x-axis is labeled with years from 2004 to 2023 and the y-axis shows the number of suicides. The second graph displays 'Hanging Suicide' trends over the same period, with fluctuations but no clear decline. The third graph illustrates 'Total Suicide' trends, showing variations with a slight decrease post-2011. Each graph includes a vertical dashed line marking December 2011, indicating a significant point in time.

Navigate back to Figure 1.

## Figure 2 Long description

A line graph showing three data series labeled as observed, fitted and counterfactual from 2005 to 2023. The x-axis is labeled 'Year and Month' and the y-axis is labeled with numerical values ranging from 0 to 500. The observed data is represented by a solid line with periodic peaks and troughs, while the fitted data follows a similar pattern. The counterfactual data is depicted with a dashed line, diverging from the observed and fitted data after an orange vertical line marking a point between 2010 and 2015. The graph illustrates changes over time, with a noticeable decline in values post-2010.

Navigate back to Figure 2.

## Figure 3 Long description

The x-axis is labeled 'Time in years' and the y-axis is unlabeled. The observed data is represented by a solid line, the fitted data by a dotted line and the counterfactual data by a dashed line. An orange vertical line marks December 2011, indicating a significant point. The graph shows a general downward trend in the data over time.

Navigate back to Figure 3.
