# Supplementary material for: Long-term effectiveness of banning highly hazardous pesticides on suicide mortality: a 12-year quasi-experimental study in South Korea
Source: Epidemiol Psychiatr Sci. 2026 May 28;35:e32. doi: 10.1017/S2045796026100705 (PMC13227128; doi:10.1017/S2045796026100705)
Supplement: Kim et al. supplementary material [file S2045796026100705sup001.docx]

**Long-term effectiveness of banning highly hazardous pesticides on suicide mortality: A 12-year quasi-experimental study in South Korea**

**Supplementary Materials**

**Contents**

eTable 1. Time series properties of monthly pesticide suicide counts

eTable 2. ARIMA model specification for pesticide suicide time series

eTable 3. Subgroup analysis of pesticide suicide following the paraquat ban

eTable 4. Comparison of count-based and rate-based ITS analysis for pesticide suicide

eTable 5. Sensitivity analysis for intervention timing

eTable 6. Sensitivity analysis for initial period length

eTable 7. Sensitivity analysis using log transformation

eTable 8. Suicide method ranking changes before and after the paraquat ban

eTable 9. Structural breakpoint analysis for gas poisoning suicide

eTable 10. Sensitivity analysis using pre-ban data only for counterfactual projection

eTable 11. A priori two-stage interrupted time series analysis of pesticide suicide, with intervention points at December 2011 (sales restriction) and November 2012 (complete ban)

eTable 12. Interrupted time series analysis for total suicide mortality

eTable 13. Exploratory difference-in-differences analysis (secondary analysis)

eFigure 1. Autocorrelation and partial autocorrelation functions for pesticide suicide time series

eFigure 2. Seasonal-trend decomposition of pesticide suicide time series

eFigure 3. Residual diagnostics for the ARIMA model

eFigure 4. Subgroup interrupted time series analyses

eFigure 5. Sensitivity analysis using pre-ban data only for counterfactual projection

eFigure 6. Exploratory difference-in-differences analysis comparing pesticide suicides with control outcomes

eFigure 7. Two-stage intervention model for pesticide suicide

eFigure 8. Interrupted time series analysis for total suicide mortality

eFigure 9. Demographic characteristics of pesticide and gas suicide decedents

eFigure 10. Structural breakpoint analysis for gas suicide

**eTable 1. Time Series Properties of Monthly Pesticide Suicide Counts in South Korea, 2004–2023**

| **Property** | **Value** |
| --- | --- |
| Observations | 240 months |
| Mean | 145.7 |
| SD | 93.9 |
| Coefficient of variation | 0.644 |
| Skewness | 0.729 |
| Box-Cox λ | 0.358 |
| Transformation applied | Square root |
| ADF test P-value | 0.01 |
| Ljung-Box test P-value | <0.001 |

Abbreviations: ADF, Augmented Dickey-Fuller; SD, standard deviation.

**eTable 2. ARIMA Model Specification for Pesticide Suicide Time Series**

| **Component** | **Specification** |
| --- | --- |
| Outcome | Pesticide suicide (monthly counts) |
| Transformation | Square root |
| ARIMA order | (1, 0, 2) |
| Seasonal order | (2, 0, 0)[12] |
| AICc | 550.19 |
| Ljung-Box P-value | 0.428 |

Abbreviations: AICc, Akaike information criterion corrected; ARIMA, autoregressive integrated moving average.

**eTable 3. Subgroup Analysis of Pesticide Suicide Following the Paraquat Ban in South Korea**

| **Subgroup** | **Parameter** | **Wald Estimate (95% CI)** | **P-value** | **Bootstrap 95% CI** |
| --- | --- | --- | --- | --- |
| **Sex** |  |  |  |  |
| Male | Level change | −0.230 (−0.967, 0.507) | 0.540 | (−3.548, 1.726) |
|  | Initial slope | −0.100 (−0.137, −0.064) | <0.001 | (−0.166, 0.004) |
|  | Later slope | 0.009 (−0.012, 0.030) | 0.395 | (−0.032, 0.035) |
| Female | Level change | −0.005 (−0.528, 0.518) | 0.985 | (−1.600, 1.107) |
|  | Initial slope | −0.071 (−0.095, −0.047) | <0.001 | (−0.114, −0.027) |
|  | Later slope | 0.008 (−0.006, 0.021) | 0.259 | (−0.016, 0.020) |
| **Age group** |  |  |  |  |
| 15–29 years | Level change | −0.515 (−1.053, 0.023) | 0.061 | (-1.650, 0.218) |
|  | Initial slope | 0.006 (−0.013, 0.024) | 0.548 | (-0.017, 0.043) |
|  | Later slope | 0.021 (0.014, 0.029) | <0.001 | (0.005, 0.030) |
| 30–49 years | Level change | 0.005 (−0.590, 0.600) | 0.986 | (-1.420, 2.836) |
|  | Initial slope | −0.046 (−0.070, −0.021) | <0.001 | (-0.106, -0.014) |
|  | Later slope | 0.026 (0.013, 0.038) | <0.001 | (0.006, 0.035) |
| 50–64 years | Level change | −0.017 (−0.503, 0.470) | 0.947 | (-1.535, 0.907) |
|  | Initial slope | −0.060 (−0.081, −0.038) | <0.001 | (-0.084, -0.001) |
|  | Later slope | 0.002 (−0.010, 0.013) | 0.790 | (-0.023, 0.015) |
| ≥65 years | Level change | −0.329 (−0.964, 0.305) | 0.309 | (-4.094, 1.020) |
|  | Initial slope | −0.107 (−0.136, −0.078) | <0.001 | (-0.165, -0.009) |
|  | Later slope | −0.015 (−0.032, 0.002) | 0.094 | (-0.043, 0.009) |
| **Region** |  |  |  |  |
| Capital | Level change | 0.195 (−0.379, 0.769) | 0.506 | (−1.254, 1.897) |
|  | Initial slope | −0.048 (−0.074, −0.021) | <0.001 | (−0.086, 0.003) |
|  | Later slope | 0.017 (0.002, 0.032) | 0.028 | (−0.010, 0.032) |
| Non-capital | Level change | −0.325 (−1.076, 0.426) | 0.396 | (−4.153, 1.412) |
|  | Initial slope | −0.114 (−0.151, −0.076) | <0.001 | (−0.180, 0.011) |
|  | Later slope | 0.004 (−0.017, 0.026) | 0.702 | (−0.036, 0.033) |
| **Occupation** |  |  |  |  |
| AFF | Level change | −0.352 (−0.964, 0.259) | 0.259 | (−1.705, 1.362) |
|  | Initial slope | −0.065 (−0.093, −0.038) | <0.001 | (−0.118, −0.019) |
|  | Later slope | −0.014 (−0.029, 0.002) | 0.081 | (−0.044, 0.002) |
| Other occupations | Level change | −0.275 (−1.074, 0.524) | 0.500 | (−1.795, 0.906) |
|  | Initial slope | −0.044 (−0.075, −0.013) | 0.006 | (−0.079, −0.004) |
|  | Later slope | 0.008 (−0.005, 0.021) | 0.211 | (−0.017, 0.020) |
| Not specified | Level change | 0.032 (−0.625, 0.689) | 0.924 | (−3.521, 1.286) |
|  | Initial slope | −0.093 (−0.124, −0.063) | <0.001 | (−0.131, 0.005) |
|  | Later slope | 0.017 (−0.001, 0.035) | 0.067 | (−0.008, 0.042) |

Abbreviations: AFF, agriculture, forestry, and fishery; CI, confidence interval.

Estimates on square root scale. Negative values indicate reduction.

**eTable 4. Comparison of Count-Based and Rate-Based Interrupted Time Series Analysis for Pesticide Suicide**

**Panel A. ITS Parameter Estimates**

| **Parameter** | **Primary: Counts (95% CI)** | **P-value** | **ASR: Wald (95% CI)** | **P-value** | **ASR: Bootstrap 95% CI** |
| --- | --- | --- | --- | --- | --- |
| Level change | −0.204 (−0.989, 0.581) | 0.610 | −0.011 (−0.046, 0.024) | 0.528 | (−0.213, 0.102) |
| Initial slope | −0.122 (−0.163, −0.082) | <0.001 | −0.005 (−0.007, −0.003) | <0.001 | (−0.009, 0.000) |
| Later slope | 0.012 (−0.012, 0.036) | 0.332 | 0.001 (0.000, 0.002) | 0.034 | (−0.001, 0.003) |

**Panel B. Model Diagnostics**

| **Outcome** | **ARIMA Order** | **Ljung-Box p** | **Shapiro-Wilk p** | **AIC** | **BIC** |
| --- | --- | --- | --- | --- | --- |
| Pesticide (counts) | ARIMA(1,0,2)(2,0,0)[12] | 0.420 | 0.144 | 549.4 | 587.7 |
| Pesticide (ASR) | ARIMA(1,0,2)(2,0,0)[12] | 0.387 | 0.329 | −512.8 | −474.5 |

Abbreviations: ASR, age-sex standardized rate (per 100,000); CI, confidence interval.

Standard population: 2010 Korean census. Ljung-Box test for residual autocorrelation (p > 0.05 indicates no significant autocorrelation). Shapiro-Wilk test for residual normality (p > 0.05 indicates normality satisfied). See Figure 3 for visualization.

**eTable 5. Sensitivity Analysis for Intervention Timing: Pesticide Suicide**

| **Intervention Timing** | **Parameter** | **Wald Estimate (95% CI)** | **P-value** | **Bootstrap 95% CI** |
| --- | --- | --- | --- | --- |
| December 2011 (Primary) | Level change | −0.204 (−0.989, 0.581) | 0.610 | (−4.497, 2.223) |
|  | Initial slope | −0.122 (−0.163, −0.082) | <0.001 | (−0.214, −0.012) |
|  | Later slope | 0.012 (−0.012, 0.036) | 0.332 | (−0.040, 0.049) |
| May 2012 (+6 months) | Level change | −1.457 (−2.230, −0.683) | <0.001 | (−7.849, 1.090) |
|  | Initial slope | −0.085 (−0.125, −0.045) | <0.001 | (−0.189, 0.099) |
|  | Later slope | 0.012 (−0.012, 0.036) | 0.317 | (−0.036, 0.049) |
| December 2012 (+12 months) | Level change | −1.383 (−2.418, −0.347) | 0.009 | (−6.790, −0.191) |
|  | Initial slope | −0.043 (−0.090, 0.003) | 0.068 | (−0.136, 0.070) |
|  | Later slope | 0.022 (−0.005, 0.050) | 0.112 | (−0.026, 0.053) |

Abbreviations: CI, confidence interval.

**eTable 6. Sensitivity Analysis for Initial Period Length: Pesticide Suicide**

| **Initial Period** | **Parameter** | **Wald Estimate (95% CI)** | **P-value** | **Bootstrap 95% CI** |
| --- | --- | --- | --- | --- |
| 6 months | Level change | 0.477 (−0.914, 1.867) | 0.502 | (−3.673, 3.537) |
|  | Initial slope | −0.398 (−0.710, −0.087) | 0.012 | (−1.123, 0.157) |
|  | Later slope | −0.009 (−0.044, 0.025) | 0.590 | (−0.067, 0.030) |
| 12 months | Level change | 0.676 (−0.453, 1.804) | 0.241 | (−2.356, 11.946) |
|  | Initial slope | −0.293 (−0.420, −0.166) | <0.001 | (−1.328, −0.103) |
|  | Later slope | −0.003 (−0.031, 0.025) | 0.817 | (−0.064, 0.038) |
| 24 months | Level change | 0.307 (−0.479, 1.093) | 0.444 | (−2.670, 8.863) |
|  | Initial slope | −0.183 (−0.231, −0.134) | <0.001 | (−0.519, −0.042) |
|  | Later slope | 0.007 (−0.015, 0.029) | 0.533 | (−0.048, 0.040) |
| 36 months (Primary) | Level change | −0.204 (−0.989, 0.581) | 0.610 | (−3.978, 2.525) |
|  | Initial slope | −0.122 (−0.163, −0.082) | <0.001 | (−0.211, −0.003) |
|  | Later slope | 0.012 (−0.012, 0.036) | 0.332 | (−0.042, 0.049) |
| 60 months | Level change | −0.540 (−1.490, 0.409) | 0.265 | (−5.073, 0.611) |
|  | Initial slope | −0.062 (−0.105, −0.019) | 0.005 | (−0.125, 0.020) |
|  | Later slope | 0.016 (−0.020, 0.051) | 0.386 | (−0.035, 0.071) |

Abbreviations: CI, confidence interval.

**eTable 7. Sensitivity Analysis Using Log Transformation for Pesticide Suicide**

| **Parameter** | **Primary: Sqrt (95% CI)** | **P-value** | **Log: Wald (95% CI)** | **P-value** | **Direction Consistent** | **Significance Consistent** |
| --- | --- | --- | --- | --- | --- | --- |
| Level change | −0.204 (−0.989, 0.581) | 0.61 | 0.009 (−0.114, 0.132) | 0.89 | No* | Yes |
| Initial slope | −0.122 (−0.163, −0.082) | <0.001 | −0.023 (−0.029, −0.018) | <0.001 | Yes | Yes |
| Later slope | 0.012 (−0.012, 0.036) | 0.33 | −0.001 (−0.004, 0.003) | 0.72 | No* | Yes |

Abbreviations: CI, confidence interval; Sqrt, square root.

*Estimates near zero; direction difference not meaningful. Log slope −0.023 = 2.3% monthly reduction.

**eTable 8. Suicide Method Ranking Changes Before and After the Paraquat Ban**

| **Method** | **Pre-ban N** | **Pre-ban %** | **Pre-ban Rank** | **Post-ban N** | **Post-ban %** | **Post-ban Rank** | **Rank Change** |
| --- | --- | --- | --- | --- | --- | --- | --- |
| Hanging | 52,038 | 48.9 | 1 | 82,723 | 50.9 | 1 | 0 |
| Pesticide poisoning | 23,183 | 21.8 | 2 | 11,779 | 7.2 | 4 | −2 |
| Jumping from height | 15,001 | 14.1 | 3 | 26,716 | 16.4 | 2 | +1 |
| Other chemical poisoning | 4,294 | 4.0 | 4 | 4,070 | 2.5 | 6 | −2 |
| Drowning | 3,345 | 3.1 | 5 | 6,201 | 3.8 | 5 | 0 |
| Gas poisoning | 3,300 | 3.1 | 6 | 23,562 | 14.5 | 3 | +3 |
| Unspecified means | 1,827 | 1.7 | 7 | 589 | 0.4 | 11 | −4 |
| Sharp object | 912 | 0.9 | 8 | 1,698 | 1.1 | 8 | 0 |
| Smoke/fire/flames | 716 | 0.7 | 9 | 880 | 0.5 | 10 | −1 |
| Sedative/psychotropic drug | 481 | 0.5 | 10 | 2,046 | 1.3 | 7 | +3 |
| Other drug poisoning | 289 | 0.3 | 12 | 1,216 | 0.8 | 9 | +3 |

Pre-ban: Jan 2004–Nov 2011. Post-ban: Dec 2011–Dec 2023. Negative rank change = less common.

**eTable 9. Structural Breakpoint Analysis for Gas Poisoning Suicide**

**Panel A. Breakpoint Detection Results**

| **Parameter** | **Value** |
| --- | --- |
| Method analyzed | Gas poisoning (X67) |
| Number of breakpoints detected | 3 |
| First breakpoint date | August 2008 |
| Paraquat ban date | December 2011 |
| First breakpoint precedes ban | Yes (by 40 months) |

**Panel B. Mean Comparison Around First Breakpoint**

| **Method** | **Pre-breakpoint Mean** | **Post-breakpoint Mean** | **Change** |
| --- | --- | --- | --- |
| Gas poisoning (X67) | 32.9 | 141.2 | +108.3 |
| Pesticide poisoning (X68) | 242.2 | 91.4 | −150.8 |

Bai-Perron method. First breakpoint preceded ban by 3+ years, suggesting no direct substitution.

**eTable 10. Sensitivity Analysis Using Pre-ban Data Only for Counterfactual Projection**

**Panel A. Pre-ban Data Counterfactual Projection**

| **Parameter** | **Value** |
| --- | --- |
| Training period | 2004.01–2011.11 (95 months) |
| Forecast period | 2011.12–2023.12 (145 months) |
| ARIMA order | (0,1,4)(0,1,1)[12] |
| Deaths averted | 11,473 |
| 95% CI | 793 to 26,700 |

**Panel B. Comparison with Primary Analysis**

| **Analysis** | **Deaths Averted** | **Difference** |
| --- | --- | --- |
| Primary (full data) | 6,106 | — |
| Pre-ban only | 11,473 | +87.9% |

Abbreviations: ARIMA, autoregressive integrated moving average; CI, confidence interval.

Wide CI expected due to long forecast horizon (145 months) exceeding training period (95 months).

**eTable 11. A Priori Two-Stage Interrupted Time Series Analysis of Pesticide Suicide, With Intervention Points at December 2011 (Sales Restriction) and November 2012 (Complete Ban)**

**Panel A. Two-stage model parameters (square root scale)**

| **Parameter** | **Estimate** | **SE** | **95% CI** | **P-value** |
| --- | --- | --- | --- | --- |
| Level₁ (Sale restriction, 2011.12) | 0.435 | 0.607 | −0.755 to 1.624 | 0.474 |
| Slope₁ (Stage 1) | −0.223 | 0.088 | −0.395 to −0.051 | 0.011 |
| Level₂ (Full ban, 2012.11) | −0.953 | 0.576 | −2.081 to 0.176 | 0.098 |
| Slope₂ (Stage 2) | 0.221 | 0.087 | 0.050 to 0.392 | 0.011 |

**Panel B. Comparison of single-stage and a priori two-stage models**

| **Model** | **AIC** | **BIC** | **Parameters** |
| --- | --- | --- | --- |
| Single intervention (primary) | 549.4 | 587.7 | 10 |
| Two-stage | 559.8 | 601.6 | 11 |
| Δ (Two-stage − Single) | +10.4 | +13.9 | +1 |

Abbreviations: AIC, Akaike information criterion; BIC, Bayesian information criterion; CI, confidence interval; SE, standard error.

The two-stage model was specified a priori based on the known policy timeline: sales restriction (December 2011) followed by complete ban (November 2012).

**eTable 12. Interrupted Time Series Analysis for Total Suicide Mortality in South Korea, 2004–2023**

| **Parameter** | **Estimate** | **SE** | **95% CI** | **P-value** | **% Change** |
| --- | --- | --- | --- | --- | --- |
| Level (immediate) | −0.086 | 0.063 | −0.209 to 0.037 | 0.170 | −8.3% |
| Slope (0–36 months) | −0.007 | 0.003 | −0.013 to −0.001 | 0.017 | −0.70%/mo |
| Slope (36+ months) | −0.004 | 0.001 | −0.007 to −0.001 | 0.006 | −0.40%/mo |

Abbreviations: CI, confidence interval; SE, standard error.

Model: ARIMA(1,0,0)(2,0,0)[12] with log transformation. Pesticide suicides ~15% of total.

**eTable 13. Exploratory Difference-in-Differences Analysis of Pesticide-Related Suicides (Secondary Analysis)**

| **Effect** | **vs. Hanging (95% CI)** | **P-value** | **vs. Non-pesticide (95% CI)** | **P-value** |
| --- | --- | --- | --- | --- |
| Immediate (level) | −0.236 (−0.622 to 0.150) | 0.231 | −0.262 (−0.620 to 0.095) | 0.151 |
| Initial (slope, 1–36mo) | −0.026 (−0.040 to −0.012) | <0.001 | −0.028 (−0.041 to −0.015) | <0.001 |
| Later (slope, 37+mo) | −0.004 (−0.008 to 0.001) | 0.088 | −0.004 (−0.008 to 0.000) | 0.032 |

Abbreviations: CI, confidence interval; DiD, difference-in-differences.

Parallel trend assumption violated. Interpret with caution. Primary analysis: Table 1.


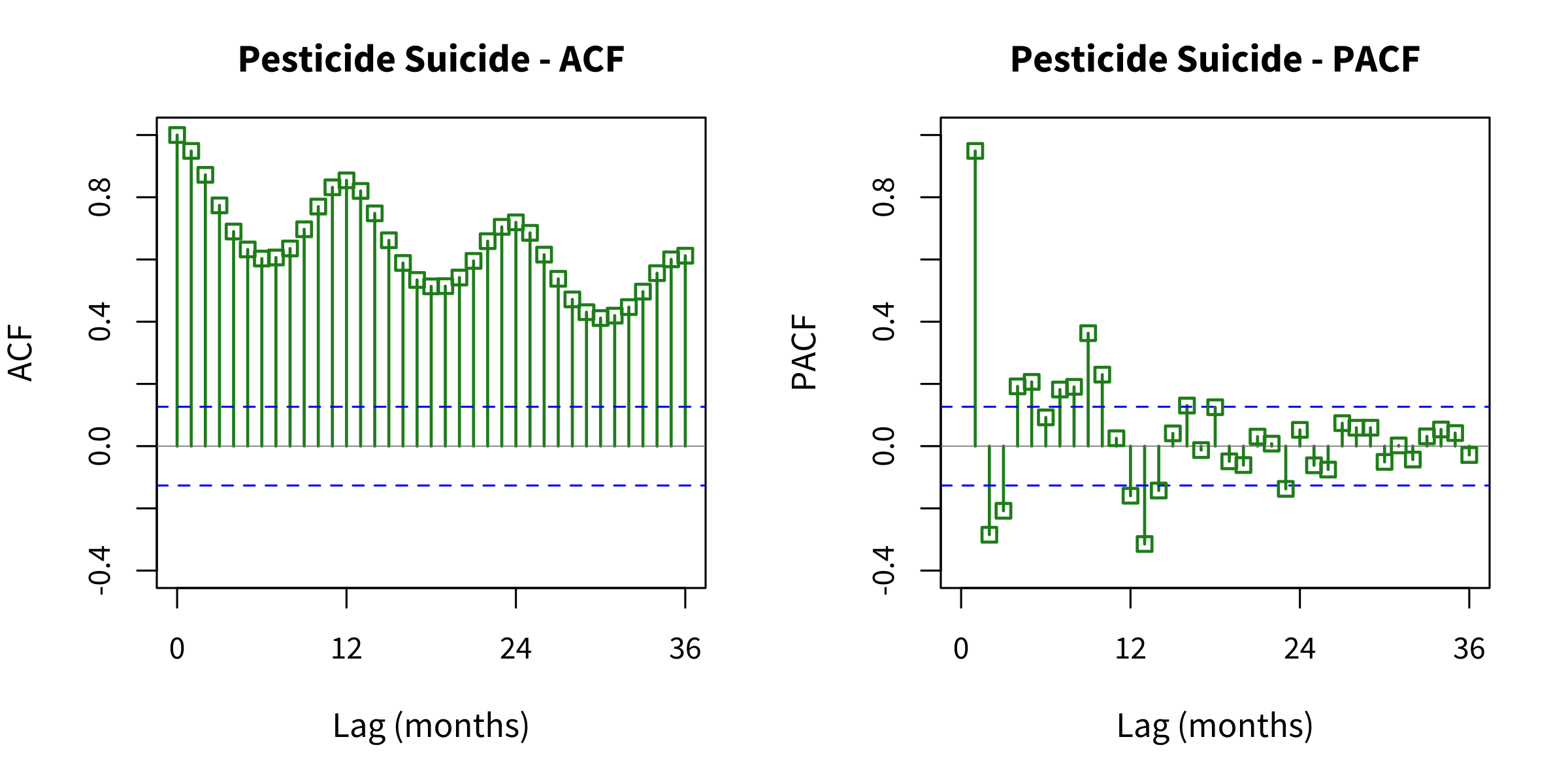


**eFigure 1. Autocorrelation and Partial Autocorrelation Functions for Pesticide Suicide Time Series**

ACF (left) and PACF (right) plots for monthly pesticide suicide counts after square root transformation. Blue dashed lines: 95% confidence bounds.


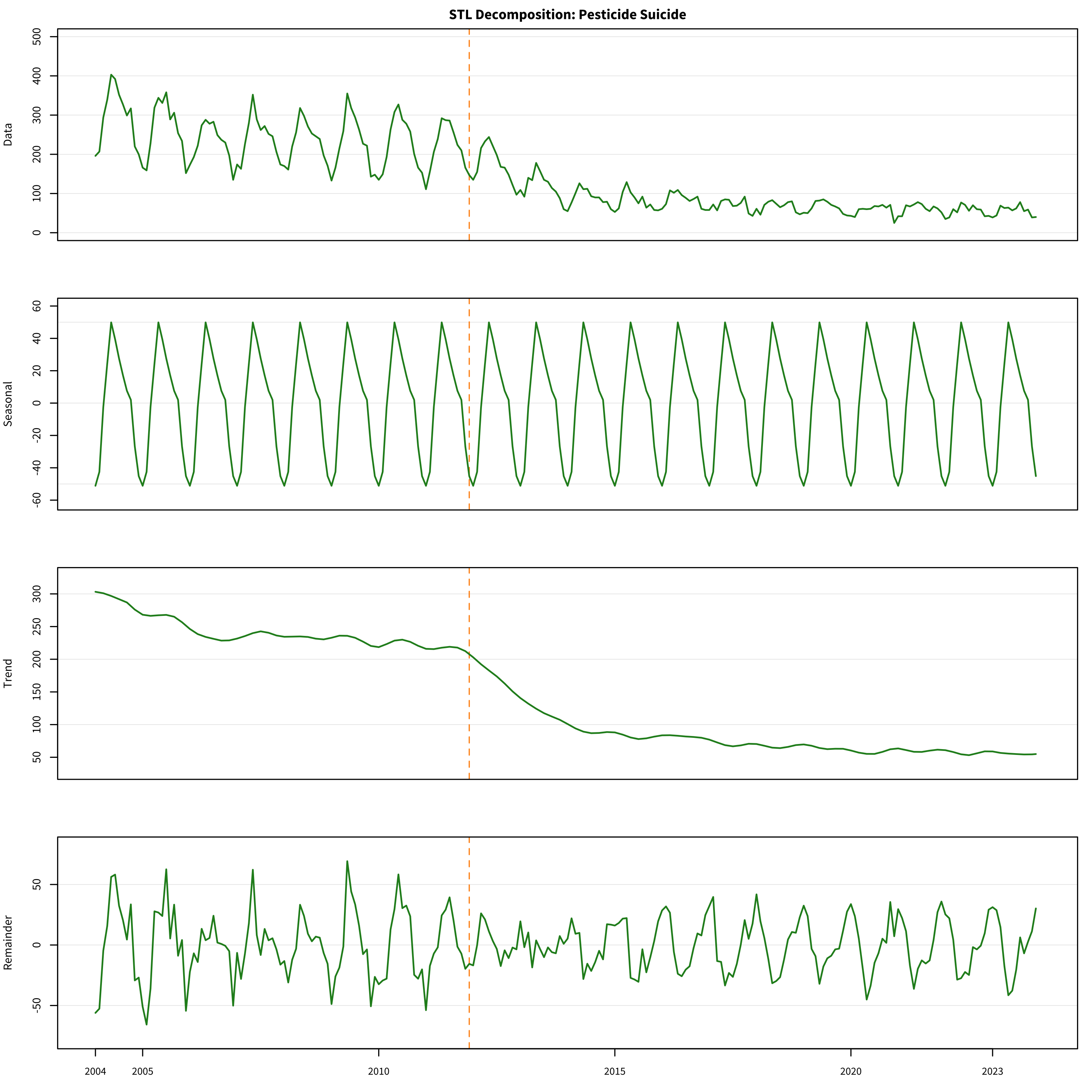


**eFigure 2. Seasonal-Trend Decomposition of Pesticide Suicide Time Series**

STL decomposition of monthly pesticide suicide counts. Panels from top: original data, seasonal component, trend component, remainder.


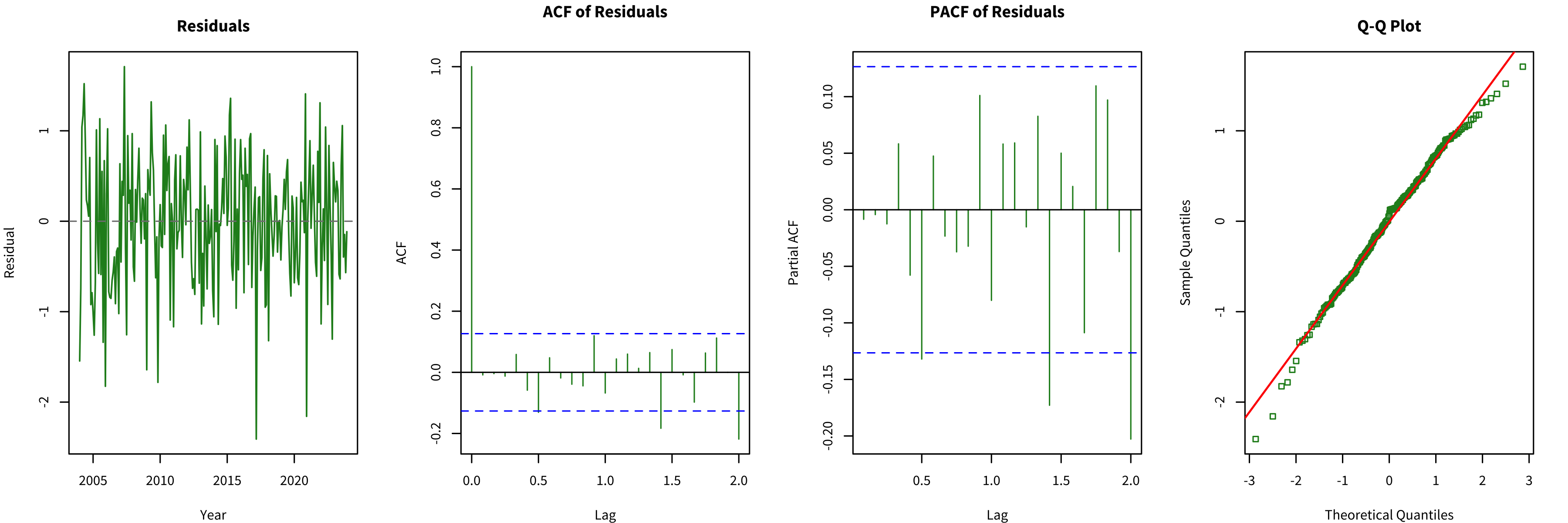


**eFigure 3. Residual Diagnostics for the ARIMA Model**

Diagnostic plots for the fitted ARIMA model. (A) Standardized residuals; (B) ACF of residuals; (C) Histogram of residuals; (D) Q-Q plot.

(A)


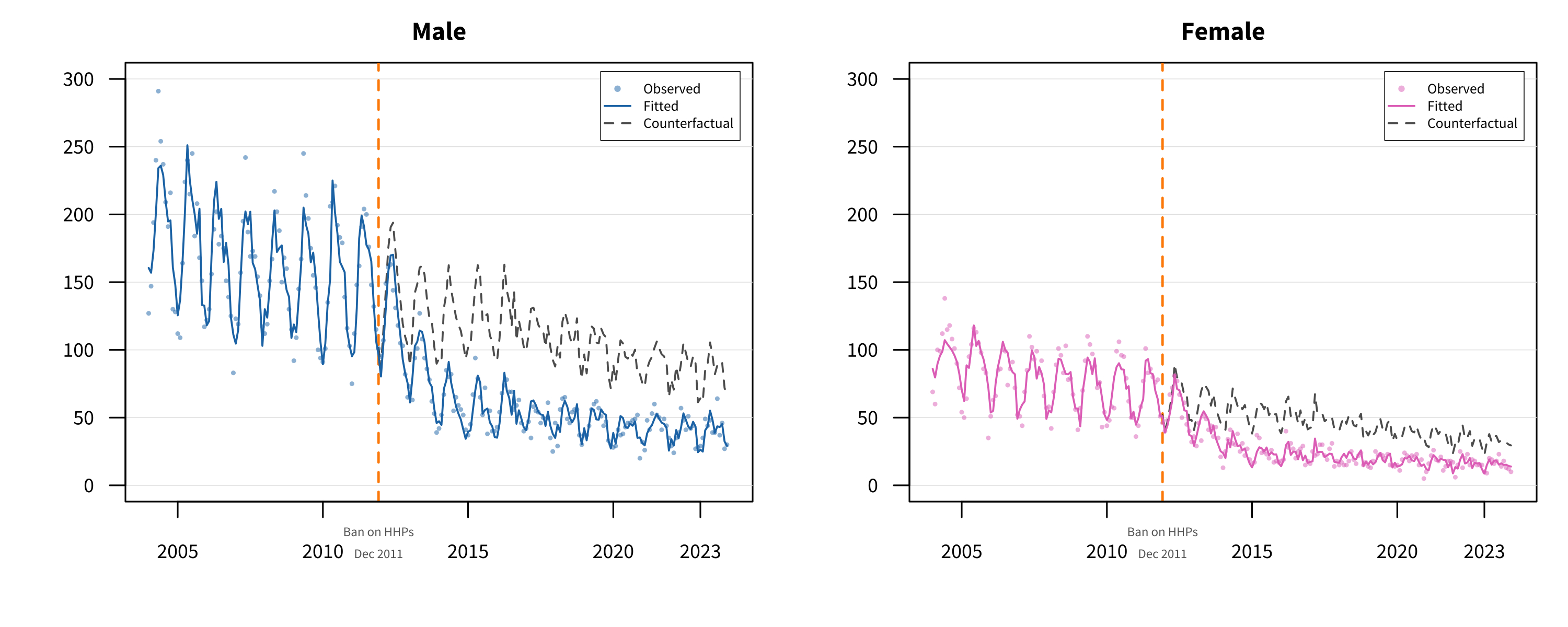


(B)


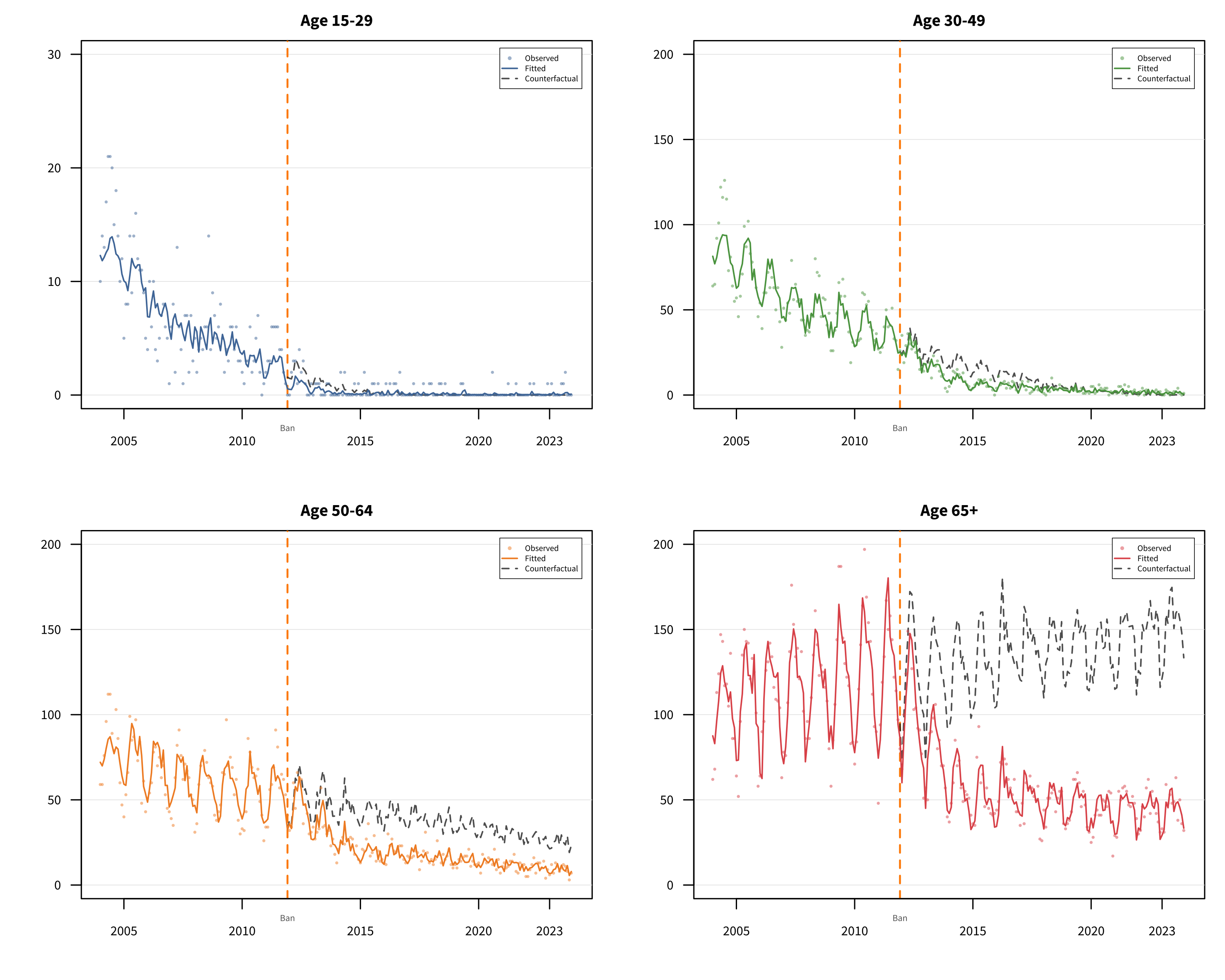


**eFigure 4. Subgroup Interrupted Time Series Analyses**

ITS analyses by demographic subgroups: (A) Sex; (B) Age group; (C) Region; (D) Occupation. Points: observed counts; solid lines: fitted values; dashed lines: counterfactual projections. Vertical dashed orange line: paraquat ban (December 2011). See eTable 3 for details.

(C)


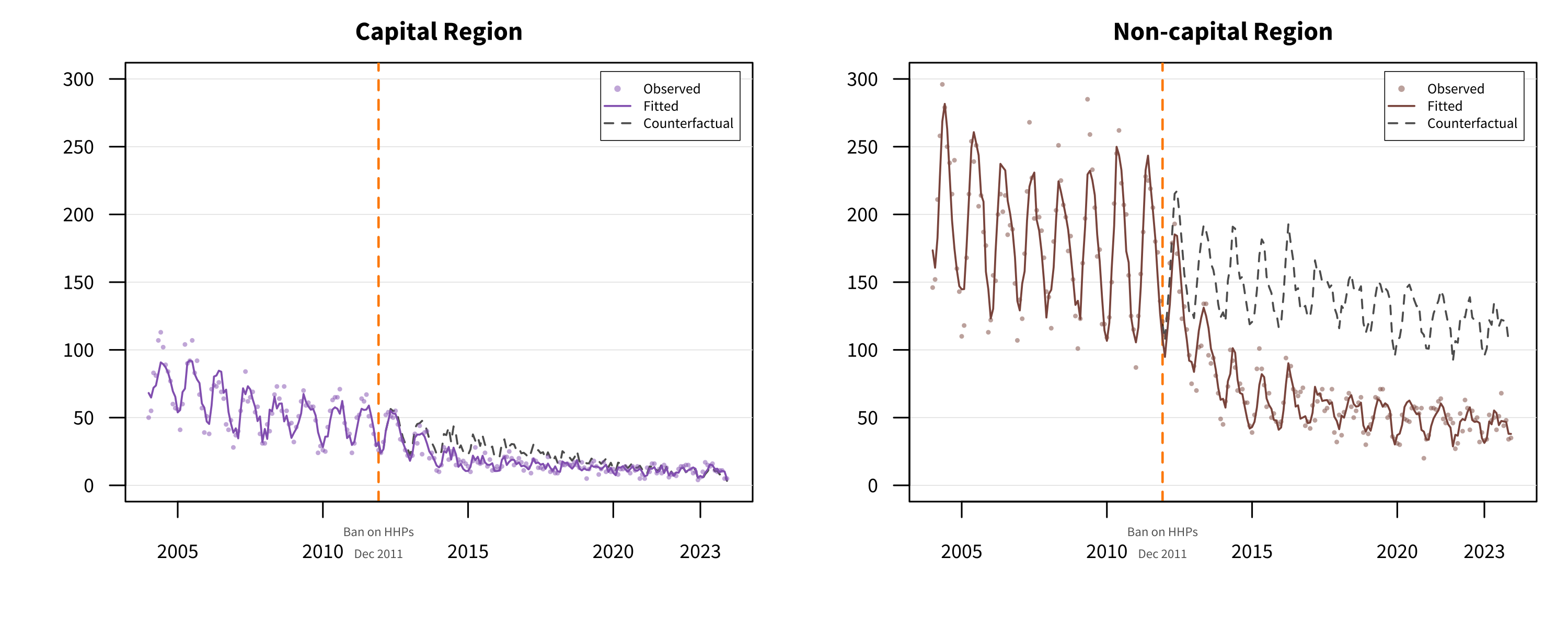


(D)


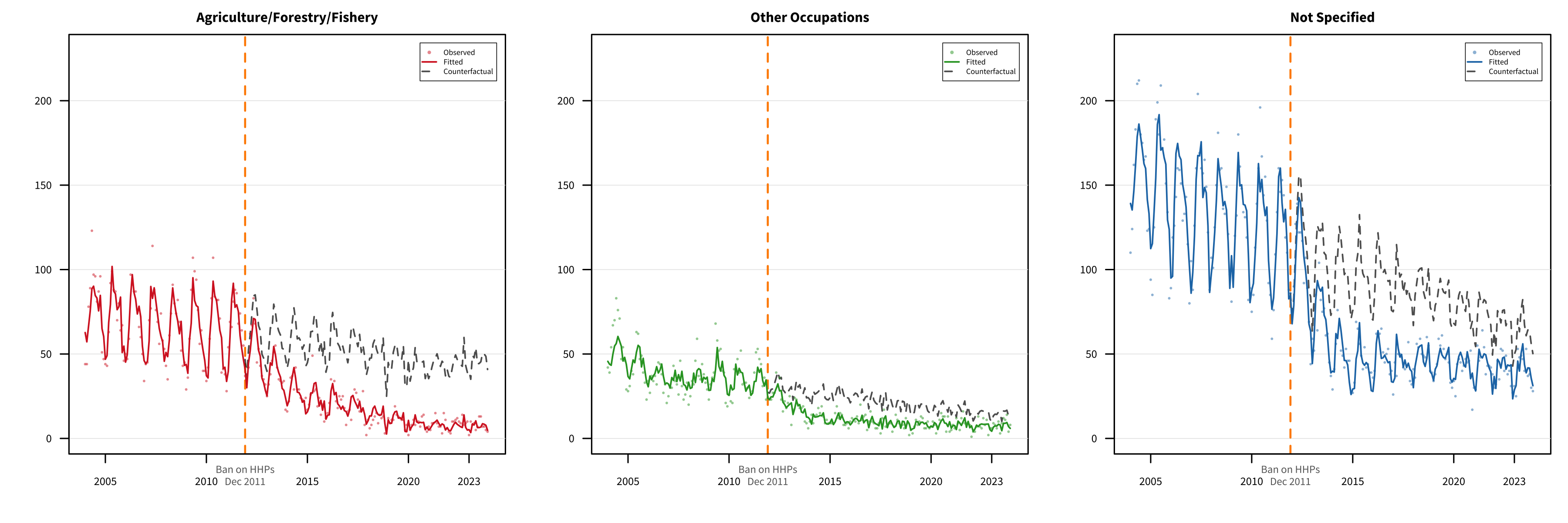


**eFigure 4. Subgroup Interrupted Time Series Analyses**

ITS analyses by demographic subgroups: (A) Sex; (B) Age group; (C) Region; (D) Occupation. Points: observed counts; solid lines: fitted values; dashed lines: counterfactual projections. Vertical dashed orange line: paraquat ban (December 2011). See eTable 3 for details.


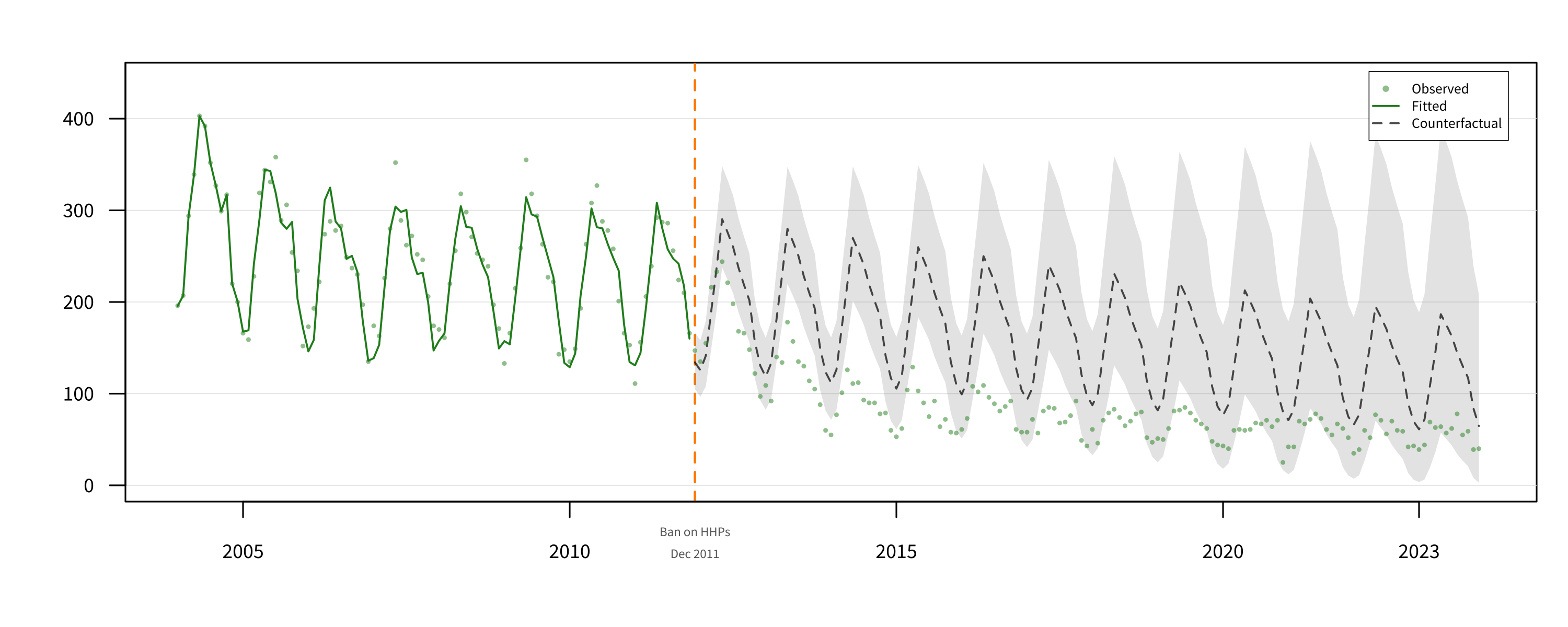


**eFigure 5. Sensitivity Analysis Using Pre-Ban Data Only for Counterfactual Projection**

Counterfactual projection using pre-ban data (2004–2011) only. Green points: observed values; green line: fitted values; gray dashed line: counterfactual forecast; gray shaded area: 95% prediction interval. See eTable 10 for details.





**eFigure 6. Exploratory Difference-in-Differences Analysis Comparing Pesticide Suicides with Control Outcomes**

Indexed monthly counts (December 2011 = 100) for pesticide suicides compared with (A) hanging and (B) non-pesticide suicides. This exploratory analysis should be interpreted with caution due to violation of the parallel trend assumption. See eTable 13 for details.

**
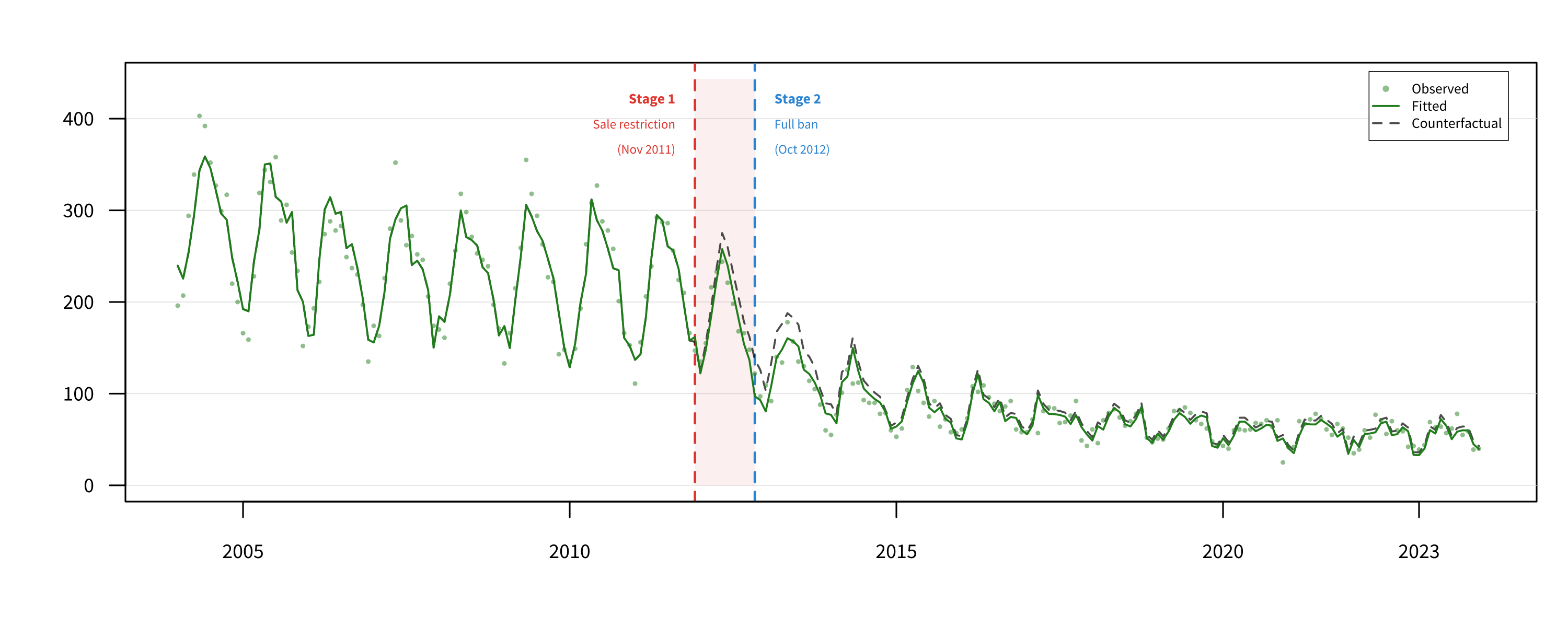
**

**eFigure 7. Two-Stage Intervention Model for Pesticide Suicide**

Interrupted time series analysis modeling the paraquat ban as two distinct intervention points: Stage 1 (sale restriction, November 2011) and Stage 2 (complete ban, October 2012). Gray points: observed monthly counts; green solid line: fitted values; gray dashed line: counterfactual projection; red dashed line: Stage 1 intervention; blue dashed line: Stage 2 intervention. The shaded region indicates the transition period between stages.

**
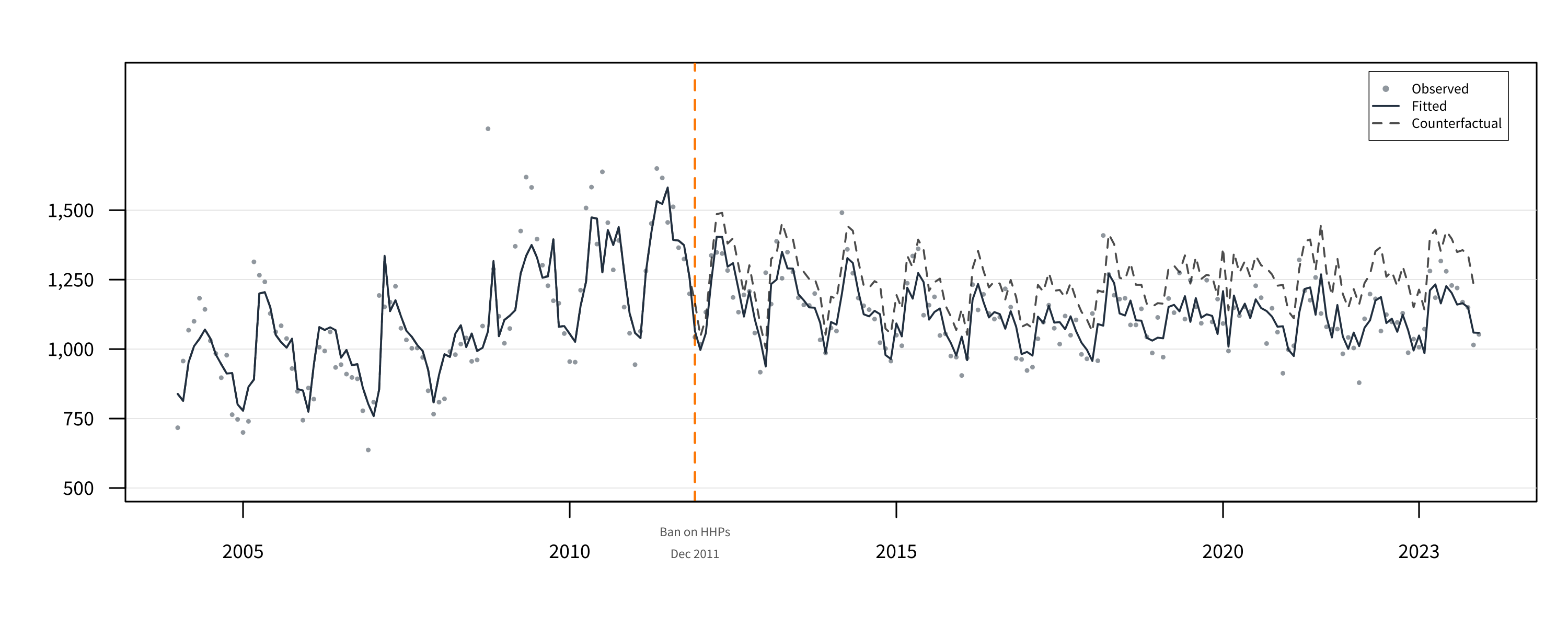
**

**eFigure 8. Interrupted Time Series Analysis for Total Suicide Mortality**

ITS analysis of overall (all-method) suicide mortality. Gray points: observed monthly counts; dark blue solid line: fitted values; gray dashed line: counterfactual projection; orange dashed line: paraquat ban (December 2011). Pesticide suicides account for approximately 15% of total suicides, limiting the detectable effect at the aggregate level.


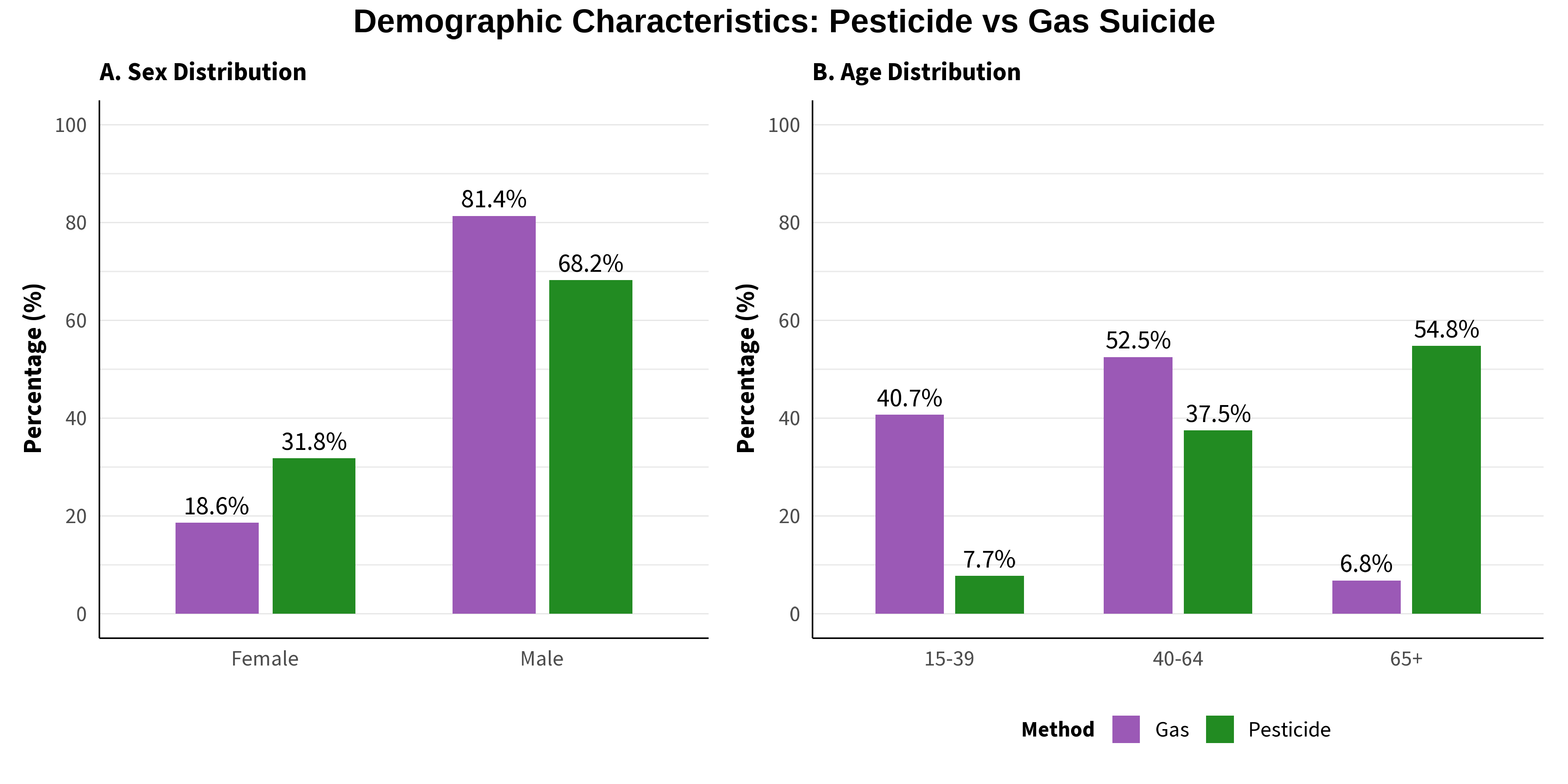


**eFigure 9. Demographic Characteristics of Pesticide and Gas Suicide Decedents**

Comparison of demographic profiles between pesticide suicide and gas suicide decedents. (A) Sex distribution; (B) Age distribution. Green bars: pesticide suicide; purple bars: gas suicide. The distinct demographic patterns support the interpretation that gas suicide increase was not primarily driven by substitution from pesticide suicide.


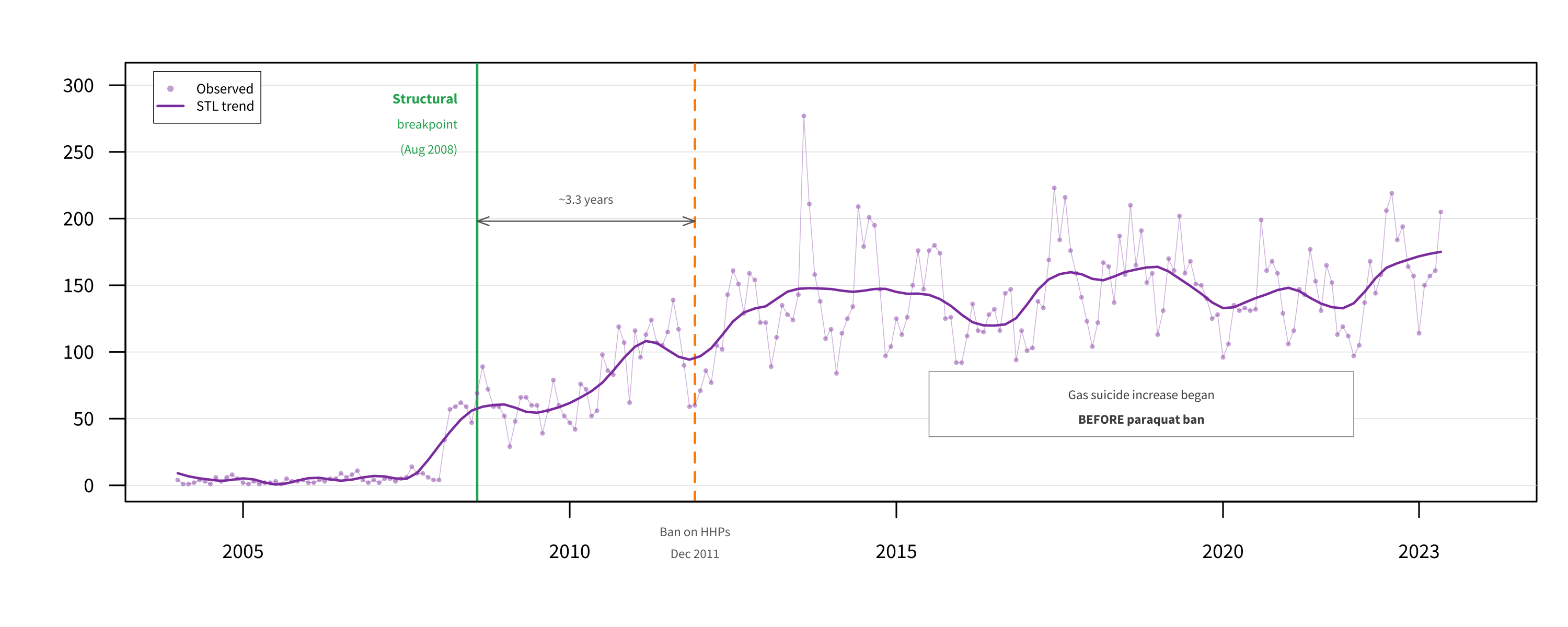


**eFigure 10. Structural Breakpoint Analysis for Gas Suicide**

Bai-Perron structural breakpoint analysis of monthly gas suicide (X67) counts with STL trend decomposition. Purple points and line: observed counts; dark purple line: trend component; green vertical line: structural breakpoint (August 2008); orange dashed line: paraquat ban (December 2011). The breakpoint occurred approximately 3.3 years before the paraquat ban, suggesting that the increase in gas suicides was driven by factors unrelated to pesticide regulation (e.g., charcoal availability, media effects following a high-profile celebrity suicide in 2008).
